# Antibiotic prophylaxis for implant placement: a systematic review of effects on reduction of implant failure

**DOI:** 10.1038/s41415-020-1649-9

**Published:** 2020-06-26

**Authors:** Andy (Seongju) Kim, Nancy Abdelhay, Liran Levin, John D. Walters, Monica P. Gibson

**Affiliations:** 1 Department of Dentistry, Faculty of Medicine and Dentistry, University of Alberta, Canada; 2 Division of Periodontology, Department of Dentistry, Faculty of Medicine and Dentistry, University of Alberta, Canada; 3 Division of Periodontology, College of Dentistry, The Ohio State University, USA

## Abstract

**Introduction** Despite excellent reviews in the past several years, the use of antibiotics as prophylaxis for implant placement remains controversial.

**Aim ** To assess the literature on the efficacy of prophylactic antibiotics prescribed prior to and immediately following implant surgery (PIFS).

**Outcomes** Whether administration of antibiotics reduced implant failure and post-operative complications.

**Design ** Databases searched were PubMed and Medline via Ovid (1946 to February 2018), Cochrane Library (Wiley) and Google Scholar.

**Materials and methods** Quality assessment, meta-analysis with a forest plot and incorporated assessment of heterogeneity. A two-tailed paired t-test was performed, analysing differences in mean failure rates between groups.

**Results** Fourteen publications were collected; 5,334 implants were placed with pre-operative antibiotics, 82 implants with antibiotics PIFS and 3,862 placed with no antibiotics. The overall risk ratio (RR) was 0.47 (95% CI 0.39-0.58), with the implant failure rates significantly affected by pre-operative intervention (Z = 7.00, P <0.00001). The number needed to treat (NNT) was 35 (95% CI 26.3-48.2). The difference between mean failure rates was statistically significant (P = 0.0335).

**Conclusion** Administering prophylactic antibiotics reduced the risk of implant failures. Further investigations are recommended to establish a standardised protocol for the proper use of antibiotic regimen.

## Key points

- Importance of antibiotic prophylaxis in preventing implant failure.
- Different types of antibiotics used for prophylaxis.
- Future considerations in antibiotic use for preventing early implant failure.

## Introduction

Antibiotic prophylaxis is standard practice for numerous surgical procedures in immunocompromised patients and in patients with significant co-morbidities.^1^ Since most patients undergoing dental implant placement are relatively healthy and do not have significant medical risk factors, the use of antibiotic prophylaxis for healthy patients has not become standard practice. Although current literature outlines long-term studies showing the efficacy of a single-dose antibiotic in reducing early implant failure, the use of antibiotic prophylaxis and choice of agent remain controversial. The primary concern involves conflicting viewpoints that antibiotic prophylaxis may or may not be as effective as traditionally believed. Xu *et al*. expressed concerns about the difficulty of limited information available to practitioners for stringent control of post-operative complications following implant procedures.^2^ Furthermore, current literature demonstrates significant variability among practitioners' antibiotic prescribing patterns, prompting the need to assess how varying interventions affect the overall success of implant operations.^3^

Dental implants generally exhibit high initial success rates, but failures occur occasionally.^3^ Implant failures during the early wound-healing period involve inflammatory breakdown at the surgical site, causing tissue scarring and poor osseointegration.^4^ One of the major disadvantages of these sequelae is the loss of hard and soft tissue at the implant site, which renders significant challenges to the surgeon for future implant placements. A retrospective analysis by Pyysalo *et al*., in 2014, demonstrated fewer numbers of early implant failures among those that were placed under antibiotic treatments.^5^

Despite these reviews, implant placement protocols presently still lack guidelines that would ensure consistent and successful post-implant wound healing.^2^

The Canadian Dental Association (CDA) has emphasised the importance of prophylactic antibiotics in managing the risk of infections associated with surgical procedures.^6^ The conflicting viewpoints are related to concerns that over-prescribing antibiotics can promote the development of antibiotic-resistant bacteria.^1^^,^^3^^,^^7^ Moreover, different regimens outlined in the literature regarding antibiotic type, dosage and the time of administration have all contributed to heterogeneity in antibiotic prescribing patterns.^8^ For instance, Ata-Ali *et al.* described antibiotic prophylaxis as necessary to provide pre-operative protection against infections and reduce the frequency of implant failures.^9^ Escalante* et al.* also supported the benefits of prophylactic antibiotic practices in implant surgery, reducing the possibility of developing post-operative infections at the surgical site.^4^ Ahmad and Saad, however, suggested that practitioners must administer prophylactic antibiotics with caution and only when determined to be appropriate, not merely as a general measure.^1^ Over-prescription of antibiotics may not only promote the emergence of antibiotic-resistant microorganisms; it could also induce toxic effects and hypersensitivity reactions.^10^

A considerable number of studies have previously reported ambivalent results, causing many to question the benefit of antibiotic prophylaxis. Limited evaluations on the importance of prophylactic antibiotics warrant further research, as the practice directly influences patient outcomes and care, associated costs, suggested lines of management and subsequent treatment planning in implant procedures. This systematic review thus aims to assess the efficacy of antibiotic use, chiefly involving administration before and immediately following dental implant surgery. We also aim to examine how current viewpoints on antibiotic practices in implant dentistry compare to the observed success rates of implants with or without the use of antibiotics.

## Materials and methods

### Search strategy

A literature search was conducted in February 2018, across the four electronic databases: PubMed and Medline via Ovid (1946 to February 2018), Cochrane Library (Wiley, February 2018) and Google Scholar. No time or language restrictions were applied to attain the maximum number of results regarding implant dentistry. Manual record searching across dental journals and other relevant databases generated additional literature more specific to the review focus. Several keywords were used during the search process, generating records pertaining to dental implants and implant failures. A detailed summary of search method and keywords is shown in Appendix 1.

### Selection of studies

The screening standard was duplicated independently by two review authors. Primary screening involved examining the title and abstract of generated records. Full-text studies that seemed to meet the criteria were included and further assessed. The two review authors independently carried out secondary screening of the remaining records, involving assessment of study methods, results and discussions. Final selection of studies was made by discussion between authors under population, intervention, comparison and outcome (PICO)-based inclusion/exclusion criteria.

### Inclusion and exclusion criteria (PICO)

The population of interest included all patients in need of implant placement and patients referred/scheduled for implant surgery. General inclusion criteria were medically and orally healthy adults (>19 years of age) who were non-smokers or light smokers (<5 cigarettes a day), had no existing periodontal disease or oral infections, and had no procedures that required antibiotic dosage before implant treatments.

Interventions were standard oral implant procedures in conjunction with a single-dose antibiotic administered pre-operatively or immediately following surgery (PIFS).

Comparison involved the effects of pre-operative antibiotics and PIFS versus the effects of no antibiotics/placebo on implant failure rates. Experimental studies directly pertaining to implant failures in the presence/absence of antibiotic prophylaxis were examined for data extraction. Included study types were randomised controlled trials (RCTs), clinically controlled trials (CCTs) and prospective/retrospective clinical studies (PCS/RCS). For meta-analysis, included study designs were RCTs and CCTs.

Outcomes of interest were implant failure rates with or without different types of prophylactic antibiotics.

Subsequently, patients were excluded if they possessed present medical conditions requiring antibiotic administration, were immunodeficient, had allergies to certain antibiotics, were non-adults (<19 years of age), were pregnant, or have had prosthetic procedures or endocarditis treatment previously (P). Treatment groups that involved long-term administration of post-operative antibiotics (duration of 2-7 days following surgery) were excluded (I). Studies without treatment interventions such as reviews, case reports and commentaries were excluded (C). Finally, records were excluded if they did not reflect the assessment of implant failures, if they had different populations of interest and if studies had different outcomes of interest (O).

### Risk of bias assessment

The risk of bias assessment for included RCTs was performed following the guidelines of the Cochrane Handbook for Systematic Reviews of Interventions.^11^^,^^12^ The criteria used in the assessment consisted of seven domains: random sequence generation, allocation concealment, blinding of assessors, blinding of outcome assessment, incomplete outcome data, selective reporting, and other bias. A study was determined to be at low risk of bias if it satisfied all of the above criteria, at moderate risk of bias if a study did not satisfy one of the above criteria, and at high risk of bias if two or more domains were not satisfied.

### Data extraction

The data extraction process was duplicated independently by two authors and then double checked between two authors to validate the gathered information. Any disagreements were resolved by discussion or consulting with other review authors.

### Data synthesis and statistical analysis

The methodology was reviewed by an independent statistician from the Strategy for Patient-Oriented Research (SPOR) networks. The meta-analysis was performed using RevMan 5.3, constructing a forest plot with I^2^ statistic to analyse variability due to heterogeneity among the gathered studies. Relative weights of included studies were expressed in percentages and the risk ratio (RR) with 95% confidence interval (CI) was computed per study or subgroup. Lastly, a two-tailed paired t-test was performed to further confer statistically significant differences between the two groups of interest. The overall effect of pre-operative antibiotic prophylaxis on implant failure rates was displayed using a bar chart.

## Results

### Gathered literature

**Fig. 1:**
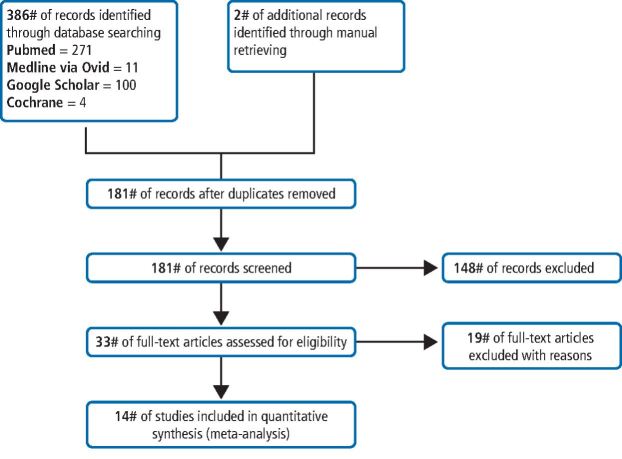
PRISMA flow diagram of literature search strategy, including identification, screening, eligibility examination and final inclusion. The number of records identified during initial search represents the sum of all papers collected through each electronic database

A summary of the search process is shown in Figure 1. The search protocol across the four databases resulted in 386 records. Manual searching across dental journals identified two additional full-text studies eligible for assessment. One hundred and eighty-one records remained after removal of duplicates and were screened via reading title and abstract, subsequently rendering 33 studies. Further evaluation employed final screening based on PICO inclusion/exclusion criteria, yielding a total of 14 relevant studies available for inclusion.

### Study design and interventions

A detailed summary of the included studies is shown in Tables 1 and 2. Data extraction identified seven RCTs,^13^^,^^14^^,^^15^^,^^16^^,^^17^^,^^18^^,^^19^ three CCTs,^20^^,^^21^^,^^22^ two pilot randomised controlled trials,^3^^,^^23^ one PCS^24^ and one RCS.^25^ All studies stated post-operative follow-up durations, as well as the outcomes of investigation. Four studies^13^^,^^18^^,^^20^^,^^25^ identified implant failures through poor osseointegration, five studies^3^^,^^19^^,^^22^^,^^23^^,^^24^ investigated pain and swelling, and the remaining five studies^21^^,^^14^^,^^15^^,^^16^^,^^17^ assessed for implant failures associated with post-operative complications and other adverse effects.

**Table 1:** The summary of specifics in each study, including follow-up durations, study designs, outcomes and interventions allowed

Author	Date of publication	Study type	Check-up durations	Outcomes of investigation	Primary intervention	Secondary intervention
**A**						
Dent *et al*.^13^	1997	RCT	Four months for mandibular anterior and six months for other sites	Failed implants: did not osseointegrate during stage I and stage II periods	a) Pre-op vs no pre-op b) Peterson recommended dose of pre-op vs no or inadequate dose of pre-op c) AHA dose of pre-op vs inadequate or no pre-op	NS
Esposito *et al*.^15^	2008	RCT	Post-operative follow-ups after one week, two weeks and four months	Post-op complications: loss of implants/prostheses and other adverse events	2 g amoxicillin one hour prior to surgery vs placebo	NS
Anitua *et al*.^16^	2009	RCT	Post-operative follow-ups after three days, ten days, one month and three months	Post-op infections, implant losses and other adverse events	2 g amoxicillin one hour prior to surgery vs placebo	NS
Laskin *et al*.^20^	2000	CCT	Thirty-six months of follow-up	Failure of osseointegration during several healing stages	a) Pre-op vs no pre-op b) Peterson recommended dose of pre-op vs no or inadequate dose of pre-op c) AHA dose of pre-op vs inadequate or no pre-op	0.12% chlorhexidine pre-op for one minute
Morris *et al*.^21^	2004	CCT	Three to five years of follow-up after surgery	Survival rates of implants and which regimen exhibited better clinical survival	a) Pre-op yes/no b) AHA-90 pre-op adequate/inadequate dose c) AHA-97 pre-op adequate/inadequate dose d) Peterson's-1990 adequate/inadequate dose	NS
Binahmed *et al*.^24^	2005	PCS	Post-op evaluation at weeks one and two, and before surgical uncovering	Pain, swelling, erythema and purulence	One hour pre-op: penicillin V intravenous or 600 mg clindamycin orally	0.12% chlorhexidine pre-op for one minute
Kashani *et al*.^25^	2005	RCS	Six months for upper jaw and three months for lower jaw	Implant survival rate: failure due to non-osseointegration	2 g phenoxymethyl penicillin one hour pre-op and same dose post-op	NS
Esposito *et al*.^29^	2010	RCT	Post-operative follow-ups after one week, two weeks and four months	Post-op complications: loss of implants/prostheses and other adverse events	2 g amoxicillin one hour prior to surgery vs placebo	NS
**B**						
El-Kholey^3^	2014	PRCT	Post-op evaluation at three days, seven days and 12 weeks	Pain, swelling, wound dehiscence and pus formation at surgical sites	Group one: 1 g single-dose oral amoxicillin one hour prior to surgery	0.12% chlorhexidine mouthwash for one minute before surgery and for five days post-operatively
Arduino^14^	2015	RCT	Followed up to six months after implant installation	Prosthetic/implant failures, adverse events and early post-operative complications	Protocol A: 2 g amoxicillin one hour before surgery with no post-operative antibiotic	NS
Nolan^18^	2014	RCT	Follow-up on day two and day seven post-operatively	Signs of post-op morbidity: swelling, bruising, suppuration and wound dehiscence. Failure defined by failed osseointegration	Test group: 3 g amoxicillin one hour prior to surgery Control group: placebo capsules one hour prior to surgery	0.2% chlorhexidine mouth rinse pre-op for at least 60 seconds
Tan^19^	2014	RCT	Examined over eight weeks after implant installation	Pain, swelling, bruising and bleeding	(i) pre-op 2 g amoxycillin one hour before surgery (ii) post-op 2 g amoxycillin immediately after surgery (iv) pre-op 2 g of placebo	0.2% chlorhexidine pre-op for one minute
Karaky^22^	2011	CCT (quasi-random)	Followed post-op at one week, one month and beginning of the prosthetic stage	Pain, wound infection, dehiscence, adverse events possibly related to antibiotics and early implant failure	Group A: 2 g amoxicillin single pre-op dose	NS
Caiazzo^23^	2011	PRCT	Follow-ups after one, two, four and eight weeks, and three months	Internal/external edema, internal/external erythema, pain, heat and exudate	Group 1: 2 g amoxicillin one hour prior to surgery Group 4: No antibiotics given	0.2 % chlorhexidine pre-op for one minute and 100 mg nimesulide twice daily for three days
Key: AHA = American Heart Association; CCT = controlled clinical trial; NS = not specified; PCS = prospective clinical study; PRCT = pilot randomised controlled trial; RCS = retrospective clinical study; RCT = randomised controlled trial

**Table 2:** The summary of specifics in each study, including number of patients/implants, antibiotic type, number of failures, success rate and observed outcomes

Author	Number of patients/implants	Antibiotic types (pre-op or PIFS)	Failed implants/total	Success rate	Findings/observed outcomes
**A**					
Dent et al.^13^	NS/1,448 (P) NS/1,193 (N)	Different regimens used by individual clinician: types, duration and dosage not stated	21/1,448 (P) 48/1,193 (N)	98.6% (P) 96.0% (N)	Significantly fewer failures of osseointegration during healing (stage I) and uncovering (stage II) when pre-operative antibiotics were used
Esposito et al.^15^	158/341 (P) 158/355 (Pl)	Pre-operative: oral amoxicillin	2/341 (P) 9/355 (Pl)	99.4% (P) 97.5% (Pl)	Placebo group experienced quadruple number of implant failures, but no significant differences observed
Anitua et al.^16^	52/52 (P) 53/53 (Pl)	Pre-operative: oral amoxicillin	2/52 (P) 2/53 (Pl)	96.2% (P) 96.2% (Pl)	Six post-op infections but no significant differences. Prophylactic antibiotics may not be necessary
Laskin et al.^20^	387/1,743 (P) 315/1,287 (Pl)	Pre-operative: Cephalosporin (13.0%) Erythromycin (7.1 %) Penicillin/derivative (69.1%) Other (10.8%)	2/52 (P) 2/53 (Pl)	96.2% (P) 96.2% (Pl)	Six post-op infections but no significant differences. Prophylactic antibiotics may not be necessary
Morris et al.^21^	NS/1,175 (P) NS/354 (N)	Regimens used varied by type, dosage and time of administration (not specified)	7/489 (P) 13/483 (Pl)	98.6% (P) 97.3% (Pl)	Higher implant success rate among patients in the pre-op group, but no significant differences
Binahmed et al.^24^	125/445 (P)	Pre-operative: intravenous penicillin V or oral clindamycin	0/445 (P)	100.0% (P)	Three cases of wound dehiscence and one minor inflammatory response. No significant difference - one pre-op dose may be sufficient in improving implant survival rate
Kashani et al.^25^	868^*^/785 (PP)	Pre-operative + PIFS: phenoxymethyl penicillin	8/785 (PP)	99.0% (PP)	Only one-day dosage recommended due to no significant difference when comparing one-day single dose vs one-week post-op administration
Esposito et al.^29^	252/489 (P) 254/483 (Pl)	Pre-operative: oral amoxicillin	7/489 (P) 13/483 (Pl)	98.6% (P) 97.3% (Pl)	Higher implant success rate among patients in the pre-op group, but no significant differences
**B**					
El-Kholey^3^	Group 1: 40/47 (P)	Pre-operative: single dose oral amoxicillin	Group 1: 0/47 (P)	100.0% (P)	Two patients showed wound dehiscence, one suffered pain and tenderness. Presumes a single pre-op dose to be generally sufficient
Arduino^14^	180/278 (P)	Pre-operative: oral amoxicillin	5/278 (P)	98.2% (P)	Six patients experienced early post-operative complications and post-operative group (not included here) lost eight implants in total. No statistically significant differences observed
Nolan^18^	Test group: 27/27 (P) Control group: 28/28 (Pl)	Pre-operative: single dose amoxicillin	Test group: 0/27 (P) Control group: 5/28 (Pl)	100.0% (P) 82.0 % (Pl)	Results showed pre-op antibiotics to be beneficial in terms of implant survival and patient comfort. No significant differences among any of the four outcomes assessed
Tan^19^	(i) 81/81 (P) (ii) 82/82 (PP) (iv) 80/80 (N)	Pre-operative: single dose amoxicillin PIFS: identical single dose of amoxicillin	(i) 0/81 (P) (ii) 0/82 (PP) (iv) 1/80 (N)	(i) 100.0% (P) (ii) 100.0% (PP) (iv) 98.7% (N)	Implant stability was slightly higher among groups treated with antibiotics, but there were no significant differences among the various groups
Karaky^22^	Group A: 73/210 (P)	Pre-operative: single dose amoxicillin	Group A†: 12/73 patients (P)	83.6% of Group A patients (P)	16.4% of Group A patients experienced early implant failure. However, no significant distinction among other groups
Caiazzo^23^	Group 1: 25/35 (P) Group 4: 25/29 (N)	Pre-operative: single dose amoxicillin	Group 1: 0/35 (P) Group 4: 2/29 (N)	Group 1: 100.0% (P) Group 4: 93.1% (N)	Pre-op group with higher success rate than the 'no antibiotic' group, but not statistically significant (small sample size)
Key: * = total number of patients in the study; the study does not specify number of patients per group † = representation by the number of patients - unable to identify number of failed/succeeded implants N = no antibiotics NS = not specified P = pre-operative antibiotics Pl = placebo PIFS = post-operative immediately following surgery PP = pre-op + PIFS					

All studies contained at least one primary test group with a pre-operative antibiotic intervention (Table 1). Two studies^19^^,^^25^ administered antibiotics PIFS and nine studies^20^^,^^21^^,^^13^^,^^15^^,^^16^^,^^17^^,^^18^^,^^19^ used either no antibiotics or placebo tablets as alternatives. Six studies^3^^,^^18^^,^^19^^,^^20^^,^^23^^,^^24^ introduced chlorhexidine mouthwash as secondary interventions and the remaining seven did not specify other interventions allowed.

### Choice of antibiotic regimen

Nine studies^3^^,^^22^^,^^23^^,^^14^^,^^15^^,^^16^^,^^17^^,^^18^^,^^19^ used oral amoxicillin, two studies^24^^,^^25^ administered penicillin derivatives or clindamycin, two studies^13^^,^^20^ did not specify the antibiotic types used during implant placements and one study^21^ used several types, with penicillin derivatives being the most common. Of the nine studies that administered amoxicillin, seven studies^14^^,^^15^^,^^16^^,^^17^^,^^18^^,^^19^^,^^22^^,^^23^ used an identical regimen (2 g single-dose amoxicillin) one hour before surgery, and the other two studies^3^^,^^18^ gave 1 g and 3 g single-dose one hour before surgery, respectively. Thirteen studies^3^^,^^13^^,^^14^^,^^15^^,^^16^^,^^17^^,^^18^^,^^19^^,^^20^^,^^21^^,^^23^^,^^24^^,^^25^^,^ provided precise numerical results of failed implants, while one study^22^ did not specify how many implants succeeded or failed (reported success rate as a proportion of patients in the test group). Lastly, only three studies^13^^,^^18^^,^^20^ reported a statistically significant difference between pre-operative groups and non-antibiotic groups. Four studies^3^^,^^14^^,^^24^^,^^25^ found no significant difference between single-dose pre-operative interventions and long-term post-operative interventions, and seven studies^14^^,^^15^^,^^17^^,^^19^^,^^21^^,^^22^^,^^23^ reported a trend favouring antibiotic prophylaxis, but no statistically significant differences.

### Risk of bias assessment

**Fig. 2:**
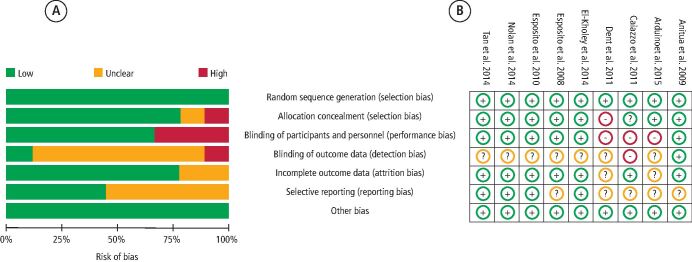
a) Overall risk of bias assessment: review authors' judgements on each risk of bias factor presented as percentages across all included studies. Following the Cochrane systematic review guidelines, studies were assessed among the seven primary domains. b) Individual risk of bias assessment: review authors' judgements about each risk of bias factor among the selected studies. Seven domains were analysed for each record and final judgements were made by discussion between authors

Two studies^13^^,^^23^ were determined to be at high risk of bias, one study^14^ at moderate risk and the remaining six studies^3^^,^^15^^,^^16^^,^^17^^,^^18^^,^^19^ at low risk of bias (Fig. 2a). All studies were considered at low risk for the domain of other potential sources of bias (Fig. 2b).

### Statistical analysis

The forest plot analysis of included RCTs and CCTs is exhibited in Table 3. Both the placebo and PIFS groups had insufficient data, hence the meta-analysis incorporated only the pre-operative antibiotic and non-antibiotic groups. The RCT group had a risk ratio (RR) of 0.38 (95% CI 0.25-0.57) for the use of pre-operative antibiotic. The CCT group had a RR of 0.57 (95% CI 0.35-0.93) for the use of pre-operative antibiotic. The RCT group exhibited low heterogeneity (I^2^ = 0%), while the CCT group exhibited medium-high heterogeneity (I^2^ = 64%). Implant failure rates were significantly affected by the difference in prophylactic measures (prophylaxis vs no antibiotic) as shown through the test for overall effect (Z = 7.00, P <0.00001; Table 3). The overall RR of 0.47 (95% CI 0.39-0.58) was followed by an absolute risk reduction (ARR) of 2.94% (95% CI 2.07%-3.80%), and the relative risk reduction (RRR) of 53%. The test for overall effect rendered low heterogeneity (I^2^ = 0%) and the number needed to treat was 35 (95% CI 26.3-48.2).

**Table 3:** Forest plot comparing the 'prophylaxis' group versus the 'no prophylaxis' group for the event of implant failure (M-H = Mantel-Haenszel test)

Study or subgroup	Antibiotic prophylaxis		No prophylaxis		Weight	Risk ratio M-H, random, 95% CI	Risk ratio M-H, random, 95% CI
	Events	Total	Events	Total			
**RCTs**							**Figure:** 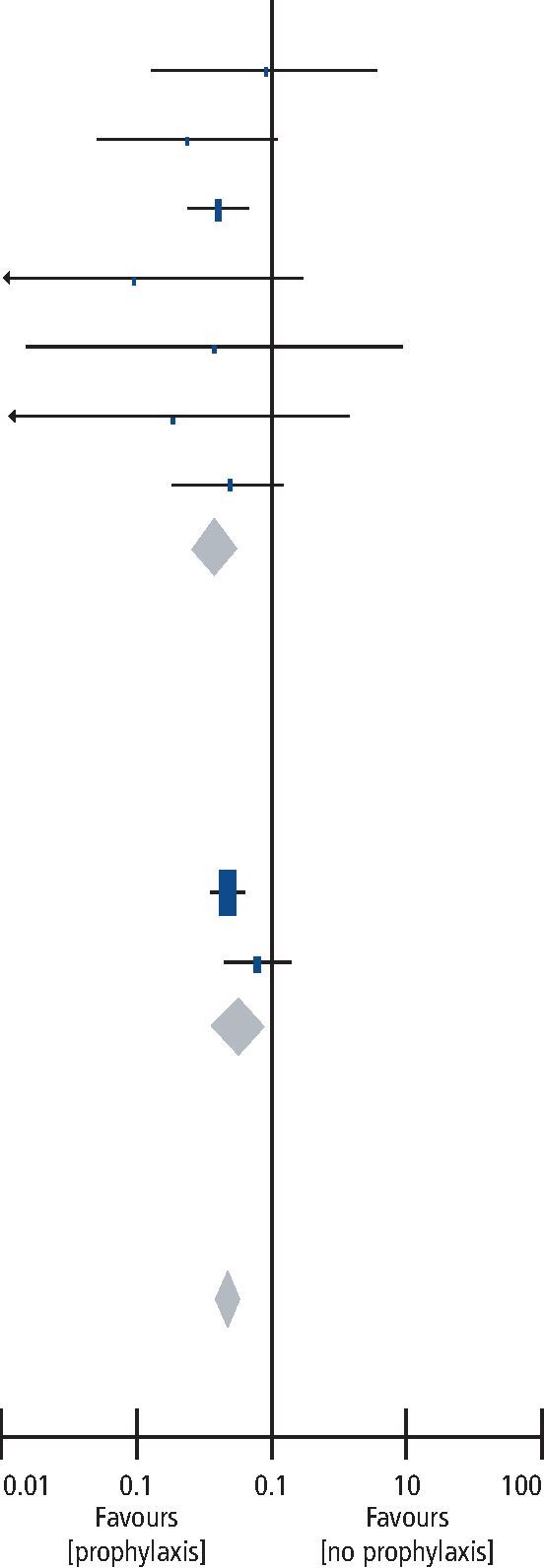
Dent (1997)^13^	21	1,448	48	1,193	16.9%	0.36 (0.22, 0.60)	
Esposito (2008)^15^	2	341	9	355	1.9%	0.23 (0.05, 1.06)	
Anitua (2009)^16^	2	52	2	53	1.2%	1.02 (0.15, 6.97)	
Nolan (2014)^18^	0	27	5	28	0.5%	0.09 (0.01, 1.62)	
Tan (2014)^19^	0	81	1	80	0.4%	0.33 (0.01, 7.96)	
Caiazzo (2011)^23^	0	35	2	29	0.5%	0.17 (0.01, 3.34)	
Esposito (2010)^29^	7	489	13	483	5.3%	0.53 (0.21, 1.32)	
**Subtotal (95% CI)**		**2,473**		**2,221**	**26.7%**	**0.38 (0.25, 0.57)**	
Total events	32		80				
Heterogeneity: Tau^2^ = 0.00; Chi^2^ = 3.24; df = 6 (P = 0.78); I^2^ = 0%							
Test for overall effect: Z = 4.71 (P <0.00001)							
**CCTs**							
Laskin (2000)^20^							
Morris (2004)^21^							
**Subtotal (95% CI)**		**2,861**		**1,641**	**73.3%**	**0.57 (0.35, 0.93)**	
Total events	122		145				
Heterogeneity: Tau^2^ = 0.08; Chi^2^ = 2.75; df = 1 (P = 0.10); I^2^ = 64%							
Test for overall effect: Z = 2.24 (P = 0.03)							
**Total (95% CI)**		**5,334**		**3,862**	**100.0%**	**0.47 (0.39, 0.58)**	
Total events	154		225				
Heterogeneity: Tau^2^ = 0.00; Chi^2^ = 7.65; df = 8 (P = 0.47); I^2^ = 0%							
Test for overall effect: Z = 7.00 (P <0.00001)							
Test for subgroup differences: Chi^2^ = 1.57; df = 1 (P = 0.21); I^2^ = 36.2%							

**Fig. 3:**
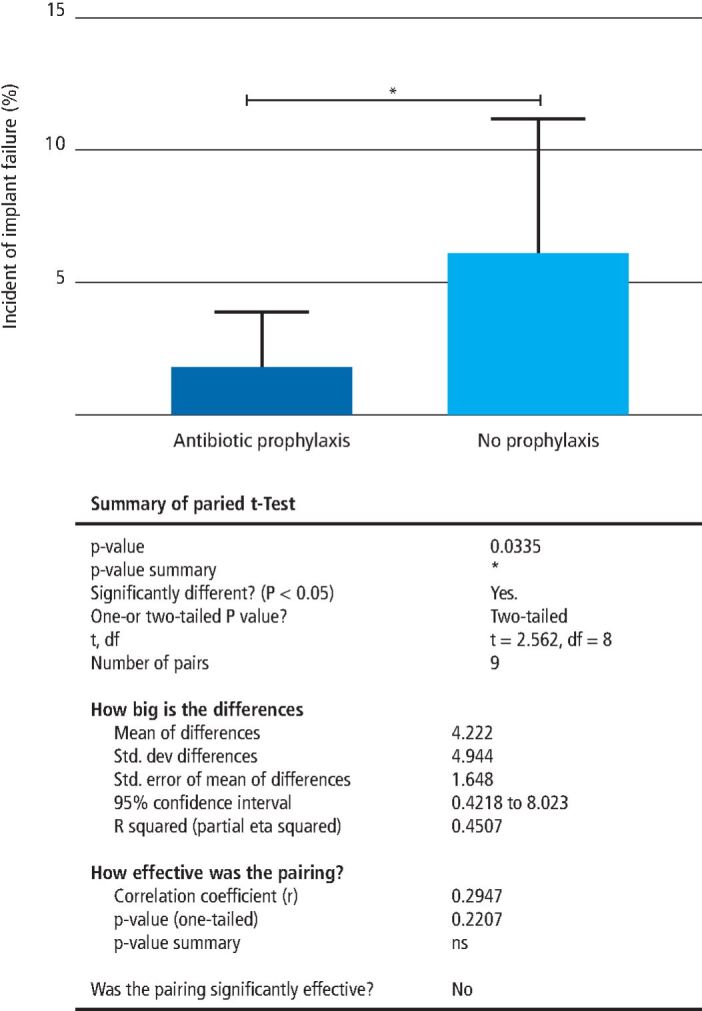
Implant failure incidence between 'antibiotic prophylaxis' and 'no antibiotic prophylaxis' groups. The 'no prophylaxis' group on average exhibited three times more implant failure incidences compared to the 'prophylaxis' group

Lastly, the two-tailed paired t-test showed significant difference in mean implant failure rates between two treatment groups, with a mean failure rate of 1.8% among the antibiotic prophylaxis patients and 6.0% in the non-prophylaxis patients (t-test, t = 2.562; df = 8; P = 0.0335; Figure 3).

## Discussion

Restricting the inclusion criteria reduces heterogeneity but limits the number of eligible studies, likely excluding papers that impart useful information.^26^ Our meta-analysis was performed accordingly, utilising not only RCTs but CCTs and observational studies to attain a wide range of data. The overall RR of 0.47 (95% CI 0.39-0.58; Table 3) for the use of pre-operative antibiotic suggested implant failures were 0.47 times as likely to occur in patients receiving antibiotic than those receiving no antibiotic. The RRR of 53% indicated pre-operative antibiotic prophylaxis reduced the risk of implant failures in the treatment group by 53%. The ARR of 2.94% is the difference in risk between the control and the treatment groups. Moreover, the number needed to treat of 35 implied one in every 35 patients will benefit from this prophylactic measure.

The medium-high heterogeneity observed between CCTs can be explained by the fact that only two studies were collected, and studies allowed various antibiotic regimen and types, sometimes not mentioned at all. Observed heterogeneity in prophylactic antibiotic choices can sufficiently render different outcomes, stressing implications for future research to identify which antibiotic regimen, type and dosage are deemed most effective. Despite low overall heterogeneity, studies varied by duration of follow-ups, secondary interventions and types of implant surgery performed (Table 1). One potential source of variation is patient heterogeneity, as patients vary in their personal idiosyncrasies, susceptibility and habits that could contribute to increased failure. Furthermore, non-standardised clinical experiences and skillsets of each practitioner may render outcome differences. Laskin *et al.* reported that implant survival rate differed by surgeons' previous clinical experiences, substantiating this possibility.^20^

It is clear that relying solely on number needed to treat and/or RRR can be misleading and result in erroneous inferences.^27^^,^^28^ Moreover, short follow-up durations tend to miss early implant failures, while extended follow-up durations overestimate total failures by incorporating those caused by other systemic conditions, environmental factors and/or personal hygiene. Taking this into account, our results still portrayed significantly fewer failures when pre-operative antibiotics were prescribed (1.8% versus 6.0%; Figure 3). Despite the limitations of our assessment in the exclusion of PIFS and placebo groups, our meta-analysis indicated significant benefits in administering pre-operative antibiotics to reduce early or late implant failures. Esposito *et al.* and Sharaf *et al.* reported a similar trend, in which a single dose of pre-operative amoxicillin significantly reduced implant failure rates.^29^^,^^30^

Additionally, Romandini *et al.* concluded that the use of prophylactic antibiotic is protective against early implant failures. However, they found insufficient evidence to recommend a specific dosage.^31^ Also, Lund *et al.* suggested that antibiotic prophylaxis gives a 2% reduction of the risk of implant loss.^32^ This investigation highlighted several problems with current antimicrobial practices in oral implantology, including: 1) use of a wide variety of prophylactic regimens; 2) lack of consistent effects of antibiotic prophylaxis; and 3) lack of standardised follow-up durations.

Myriad antibiotic choices were available to practitioners, often involving three or more types and dosages applicable to each intervention. Lack of consensus between practitioners concerning distinct antibiotic prescribing patterns warrants the need to facilitate calibration to enhance treatment homogeneity. Current literature leans towards the use of oral amoxicillin, where roughly 80% of the respondents used amoxicillin for patients not allergic to penicillin, according to a study conducted in the UK.^7^ The study also revealed the types of pre-operative antibiotics given to patients allergic to penicillin: 48.1% administered clindamycin, 19.2% metronidazole and 4.8% erythromycin.^7^ Other records further portrayed the predominance of amoxicillin uses,^33^ shown through the nine studies^3^^,^^14^^,^^15^^,^^16^^,^^17^^,^^18^^,^^19^^,^^22^^,^^23^ included in this review as well. Some clinicians, however, chose other alternatives such as clindamycin, Keflex, cefazolin and other penicillin derivatives.^34^ Pyysalo *et al.* reported phenoxymethylpenicillin (72.2%), followed by amoxicillin, cephalexin, roxithromycin and clindamycin.^5^ Ahmad and Saad also mentioned clindamycin as an alternative to amoxicillin and penicillin derivatives.^1^ In contrast, Escalante *et al.* proposed azithromycin as an effective alternative prophylactic antibiotic for patients allergic to penicillin.^4^

Although 2 g of amoxicillin is the most commonly administered pre-operative dose among clinicians, the literature is unclear on which antibiotic exhibits the most effective result. As mentioned earlier, the American Heart Association recommends only a single-dose pre-operative antibiotic prophylaxis in most of the cases, while some patients with high-risk heart diseases may require additional post-operative doses to prevent secondary bacteraemia following dental procedures.^4^^,^^10^ In the vast majority of cases, evidence suggests that providing antibiotics pre-operatively rather than post-operatively following routine dental implant placement is the protocol of choice.^7^ Only in a small number of cases, the presence of a disease may increase the risk of bacteraemia associated with these routine activities accordingly; in this condition, the benefits of post-operative antibiotics are emphasised despite the recommended guidelines.^32^

In addition, most antibiotic interventions varied by their duration, sometimes exceeding the duration needed to prevent post-operative complications. Prolonged administration of antibiotics may contribute to the emergence of resistant bacteria.^35^ Incorrect use of antibiotics with little consideration of repercussions increases the risk of developing resistance and other adverse side effects, and ultimately devalues the efficacy of antibiotic prophylaxis.^36^ One of the important factors that should be considered is evaluating the cost of the antibiotics to the desired outcomes. Due to the increase of healthcare costs and limited resources, policymakers and healthcare payers are also concerned about the cost-effectiveness of the excessive prescription of antibiotics. Hence, more studies of economic evaluation to assess the cost-effectiveness of antibiotics by exploring whether antibiotic treatment makes a sufficient contribution to health to justify its costs should be explored more in the future.^37^ These factors still suggest that antibiotics should be prescribed with caution and for the shortest duration possible to achieve the desired outcome.

Our study shows the benefits of pre-operative antibiotics in reducing dental implant failure. However, there is no standardised guide to the use of antibiotics in dental implant surgery, accentuating the need for further investigations to identify the most effective antibiotic regimen for reducing implant failures.

**Table:** **Appendix 1** Searched databases, keywords of relevance and total results identified during initial investigation

Database	Search methods	Results
PubMed/Medline	1. ("dental implants"[MeSH Terms] OR ("dental"[All Fields] AND "implants"[All Fields]) OR "dental implants" [All Fields] OR ("dental"[All Fields] AND "implant" [All Fields]) OR "dental implant"[All Fields])	271/11
	2. (("antibiotic prophylaxis"[MeSH Terms] OR ("antibiotic" [All Fields] AND "prophylaxis"[All Fields]) OR "antibiotic prophylaxis"[All Fields] OR Antibiotic Premedication [All Fields] OR antimicrobial agents[All Fields]))	
	3. ((infection* OR "wound healing"[MeSH Terms] OR ("wound"[All Fields] AND "healing"[All Fields]) OR "wound healing"[All Fields]) OR (implant[All Fields] AND ("integration"[All Fields] OR "integration"[All Fields]) OR osseointegrat*) OR fail* OR (("antimicrobial"[All Fields]) AND ("prevention and control "[Subheading] OR ("prevention"[All Fields] AND "control" [All Fields]) OR "prevention and control"[All Fields] OR "prophylaxis"[All Fields])))	
	4 1 AND 2 AND 3	
	5 4 AND Animals[Mesh:noexp]	
Cochrane Library	Dental AND implant* AND antibiotics	4
Google Scholar	Antibiotic prophylaxis and dental implant failures (first ten pages)	100
**Total**		**386**

## Conclusion

Based on our meta-analysis and statistical results, there is adequate evidence to suggest that a single-dose antibiotic prescribed pre-operatively may reduce the occurrence of implant failures. Administering prophylactic antibiotics before implant surgery can provide significant benefits to patients receiving the treatment. The observed overall reduction in risk provides support for use of prophylactic antibiotics in implant dentistry.
